# Piezoelectric Driving of Vibration Conveyors: An Experimental Assessment

**DOI:** 10.3390/sl30709174

**Published:** 2013-07-17

**Authors:** Domingos Alves Rade, Emerson Bastos de Albuquerque, Leandro Chaves Figueira, João Carlos Mendes Carvalho

**Affiliations:** 1 School of Mechanical Engineering, Federal University of Uberlandia, Campus Santa Monica, Building 1M, Av. Joao Naves de Avila 2121, Uberlandia, MG 38408-100, Brazil; E-Mails: leandrochavesfig@yahoo.com.br (L.C.F.); jcmendes@mecanica.ufu.br (J.C.M.C.); 2 Petroleo Brasileiro S.A., Rua General Canabarro, 500, Maracana, Rio de Janeiro, CEP 20271-205, Brazil; E-Mail: emersonba@petrobras.com.br

**Keywords:** vibratory feeder, vibratory conveyor, automation, piezoelectricity

## Abstract

Vibratory feeders or vibratory conveyors have been widely used for the transport and orientation of individual parts and bulk materials in many branches of industrial activity. From the designer's standpoint, the current endeavor is to conceive efficient vibratory feeders, satisfying constraints of power consumption, vibration transmission and noise emission. Moreover, the interest in the reduction of maintenance cost is always present. In this context, this paper investigates experimentally the concept of vibratory conveying based on the use of piezoelectric materials for motion generation. A small-size prototype of a linear conveyor, in which lead-zirconate-titanate (PZT) patches are bonded to the resilient elements, is described. One of the main design goals is that the prototype is intended to be fed directly from the electric network, aiming at avoiding the use of electronic equipment for driving. To comply with this feature and, at the same time, enable to adjust the transport velocity, a mechanical device has been conceived in such a way that the first natural frequency of the conveyor can be changed. It is shown that the transport velocity is determined by the proximity between the excitation frequency and the first natural frequency of the conveyor. The experimental tests performed to characterize the dynamic behavior of the prototype are described and the range of transport velocities is determined.

## 1. Introduction

**Figure 1.**
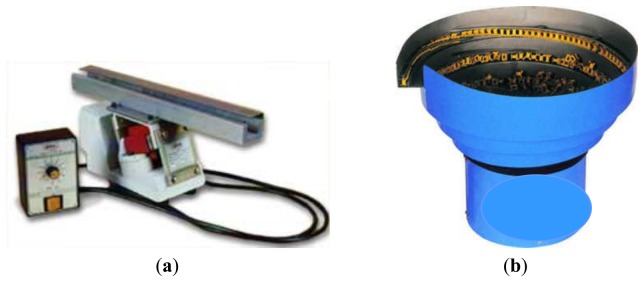
The two main types of vibrating feeders: (a) linear feeder; (b) bowl feeder.

Vibratory feeders are among the most ancient types of mechanical systems used for transportation and orientation of individual parts and bulk materials in many branches of industrial activity [1]. Compared to other transport systems, they present a number of advantageous characteristics such as precision, productivity, flexibility and reliability. Regarding the trajectory followed by the transported material, there are two main types of vibratory feeders: linear feeders and bowl feeders, which are both illustrated in Figure 1.

The three main components of a vibratory feeder are:

(a) a driving (or excitation) mechanism, which is responsible for motion generation;
(b) a track, which is excited by the driving mechanism, and transmits the motion to the transported material which moves along it;
(c) an elastic support, which connects the track to the conveyor's base and provides the necessary mechanical flexibility for adequate transmission of motion from the driving mechanism to the track.

**Figure 2.**
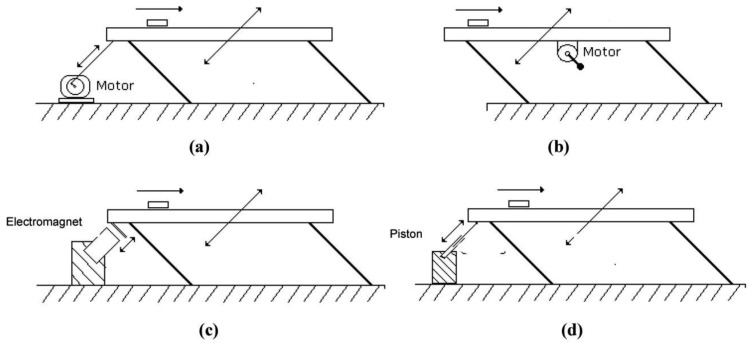
Traditional types of excitation mechanisms of vibratory feeders. (a) crank mechanism; (b) unbalanced mass; (c) electromagnetic; (d) pneumatic/hydraulic (the arrows indicate directions of motion).

These components are illustrated in Figure 2, in which the most common types of driving mechanisms are illustrated for a linear vibratory feeder. Although the traditional types of excitation mechanisms are widely used, they present some practical shortcomings. The first two types indicated in Figure 2 are prone to frequent maintenance interventions owing to the wear of moving parts in contact with each other. Electromagnetic excitation is limited by the magnitude of the excitation forces, since high forces require large and heavy electromagnets. Moreover, it has been demonstrated by Martins [2] that it becomes difficult to predict the actual motion generated by electromagnetic excitation as the electromagnetic forces are the result of nonlinear interactions between a metallic moving part and the electromagnetic field. Pneumatic and hydraulic driving systems, on their turn, are in general heavy and costly, being more adequate for large transportation systems. Additionally, high levels of noise and vibrations transmitted to the surroundings are common features of many traditional types of industrial vibratory conveyors.

This paper addresses an alternative excitation mechanism for vibratory feeders, which is based on the use of piezoelectric actuators for motion generation. Variants of this concept have been the subject of some patents [3,4] and some models are commercially available [5,6]. More recently, a study comprising the dynamic modeling and experimental assessment of a piezoelectric vibration feeder based on parallel bimorph beams has been presented [7].

The authors have been contributing to the diffusion of the concept of piezoelectric vibration transportation in industry by proposing some improvements, such as the introduction of a mechanical tuning device, which enables to adjust the transportation velocity by feeding the conveyor directly from the electrical network. This makes unnecessary the use of expensive power amplifiers as that used in [5] or electronic circuits to vary the frequency of vibration excitation.

When compared to traditional excitation mechanisms, piezoelectric driving can provide some advantages such as:

(1) they enable increased flexibility in terms of the form and amplitude of excitation (not necessarily harmonic), which can be easily prescribed through the input voltage signal. As a result, they enable a certain degree of motion control of the transported material by a proper choice of the characteristics of the excitation voltage (amplitude, frequency and waveform).
(2) since no contact between moving parts occurs, the negative consequences of contact, such as wear, noise emission, energy consumption and maintenance interventions, are avoided.

A particular point of interest is related to the first feature described above. Clearly, it is perfectly feasible to vary the characteristics of the feeding voltage by using a signal generator connected to a voltage amplifier. However, such implementation requires a certain amount of electronic equipment and, as a result, increased costs. Hence, aiming at providing the designers and operators with the possibility of controlling the transport velocity for different operation requirements and transported materials, and, at the same time, avoiding the use of costly electronics, it is proposed in this study to feed the piezoelectric actuators directly from the electrical network and introduce, in the conveyor's structure, a mechanical stiffness regulator which enables to change the value of its first natural frequency. As will be demonstrated, the proximity of the value of this frequency to the frequency of the sinusoidal feeding signal determines the transportation velocity.

In the remainder, an experimental study carried out on a small prototype of linear feeder in which piezoelectric patch actuators are fully integrated to the resilient elements is described.

## 2. Prototype of a Linear Vibrating Feeder with Piezoelectric Driving

**Figure 3.**
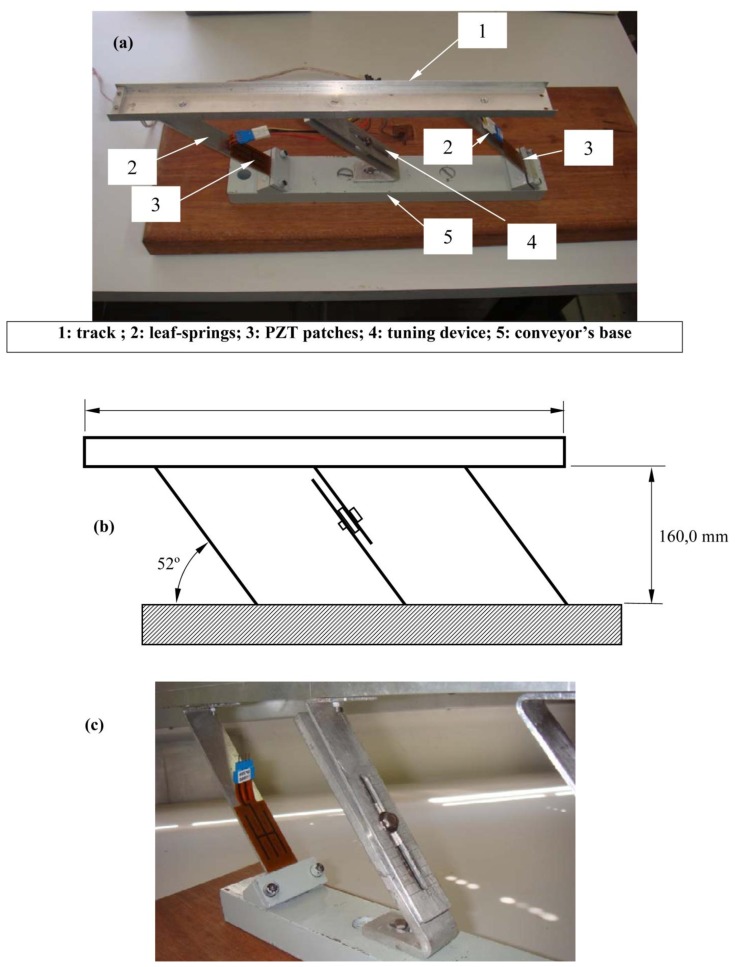
(a) Photograph of the piezoelectric vibratory feeder; (b) Main dimensions of the prototype; (c) detail of the tuning device.

Several possible forms of exploring the piezoelectric effect for generating the motion in both linear and bowl vibratory feeders have been considered by the authors [8]. Among them, the configuration illustrated in Figure 3(a,b) has been chosen for constructing a prototype for the experimental study. In this configuration, piezoelectric patches are bonded to one side of each of the two leaf-springs that connect the track to the fixed base of the conveyor. The electrical wiring is made in such a way that both piezoelectric patches are connected in parallel, thus being fed simultaneously from the electrical network to produce bending deformation of the leaf-springs.

The leaf-springs are rigidly connected to the base and track through bolts. As for the tuning device, depicted in Figure 3(c), it is bolted to the track and connected to a much stiffer folded plate by means of a sliding bolt-washer-nut assembly. On its turn, the folded plate is bolted to the conveyor base.

Basically, the tuning device consists of a third leaf-spring clamped to the track, whose effective length can be changed by moving the position of the bolt-washer-nut assembly. From the mechanical point of view, this corresponds to changing the equivalent stiffness of the suspension system formed by the three parallel leaf-springs. The tuning device possesses a ruler with a 0.5 cm resolution which is used to locate the bolt. Thus, the larger the numerical indication of the bolt position, the more flexible the suspension is.

The main interests in adopting this configuration are the ease of construction and the amplification of the motion generated by the piezoelectric actuators through the bending of the leaf-springs. The main characteristics of the components of the prototype are given in Table 1. Additionally, it should be pointed-out that the leaf springs and the tuning device are all inclined of a same angle (approx. 52 degrees).

**Figure 4.**
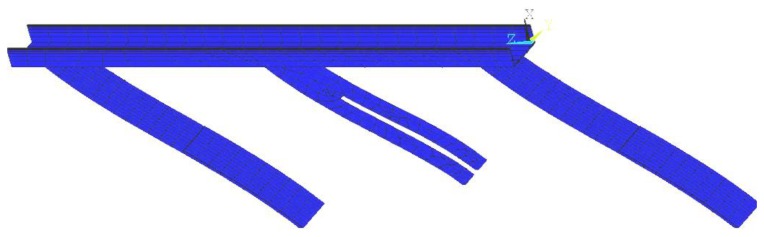
Illustration of the first mode shape of the prototype calculated by finite element analysis.

It is obvious that the characteristics of the material motion along the track are strongly dependent on the motion of the track itself which, on its turn, depends on the characteristics of the voltage signal applied to the piezoelectric actuators. Also, as is the case for any mechanical structure, the vibration response of the conveyor is determined by the interaction between the excitation forces and its modal characteristics. In the case of the linear vibratory feeder, it was found that the primary motion of the track is mainly governed by the first vibration mode, in which only the leaf-springs bend in phase and the track moves as a rigid-body, without any noticeable elastic deformation. This vibration mode-shape, which has been computed by finite element analysis, is illustrated in Figure 4.

As mentioned before, the PZT patches can, in principle, be fed with any type of voltage signal provided that this signal can be generated by appropriate electronics. However, considering practical industrial applications and the need of cost reduction, the interest is to have the minimum amount of equipment. To comply with these constraints, it was found convenient to feed the PZT directly from the electrical network (thus with a sinusoidal wave of fixed frequency) and to incorporate into the feeder a mechanical tuning device which would enable to change arbitrarily, within a certain range, the value of the first natural frequency of the structure and thus modify the vibration amplitude accordingly. Such modification is expected to influence the characteristics of the motion of the transported material, mainly the transport velocity.

## 3. Experimental Procedures and Results

The experiments were performed in two phases. In the first one, vibration tests were carried-out to identify the values of the first natural frequency of the conveyor's structure for different positions of the bolt along the tuning device. The main interest was to verify the range of values within which the natural frequency could be set.

For each position of the bolt along the tuning device, the first natural frequency was identified by acquiring frequency response functions (FRFs) which were obtained by applying a random voltage signal to the piezoelectric actuators and measuring, with a piezoelectric accelerometer, the time response of the track in the horizontal direction. The accelerometer, which is not seen Figure 3, is a very lightweight sensor (PCB model 352C22, weighing 0.5 grams) placed in one of the extremities of the track, in such a way as to enable the measurement its acceleration in the horizontal direction. The excitation voltage and acceleration signals were recorded and processed for obtaining the FRFs according to standard signal processing procedures and Fast Fourier Transforms implemented in a two-channel frequency analyzer [9].

**Figure 5.**
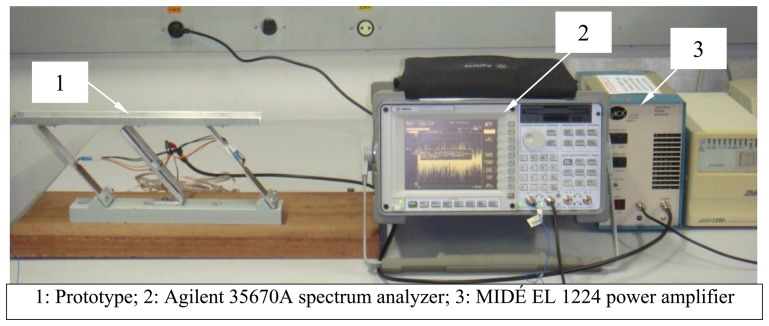
Illustration of the experimental setup.

The random excitation signal was generated by the frequency analyzer in the range [−5 V; +5 V] and amplified 20 times by a power amplifier, before being applied to the piezoelectric patches. The components of the experimental setup are shown in Figure 5.

**Figure 6.**
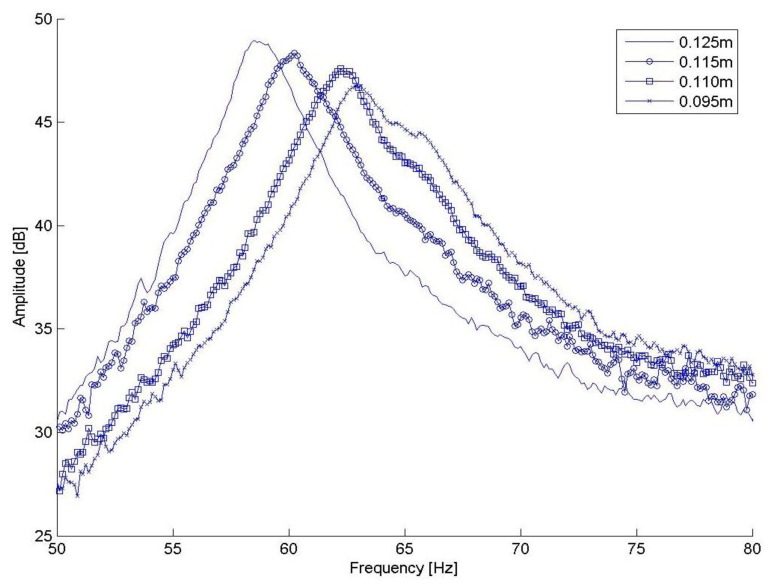
Amplitudes of the FRFs for different positions of the bolt along the tuning device.

The FRFs were averaged over 10 samples aiming at reducing the influence of experimental uncertainties which propagate from time domain measurements to the estimated FRFs. The amplitudes of some of the FRFs obtained for different positions of the bolt along the tuning device are illustrated in Figure 6. Table 2 presents the values of the first natural frequencies for each position of the tuning device, which are given by the values of the excitation frequency corresponding to the peaks of the FRFs amplitudes shown in Figure 6. The tests have shown that the tuning device enables the first natural frequency of the conveyor to be adjusted in the range from 53.5 Hz to 77.3 Hz.

The second series of experiments was carried-out to verify the performance of the vibratory conveyor when transporting isolated parts. With this aim, the piezoelectric actuators were connected to the electrical network (110 V, 60 Hz) and a single nut was used as the transported part. By measuring the time elapsed during the motion of the nut between two marks made in the track, 20 cm apart from each other, the mean transport velocities were computed for a set of different positions of the bolt along the tuning device. For each position of the bolt, four measurements were made to characterize the transport velocities in terms of mean values and coefficient of variation (defined as the ratio between standard deviation and mean value), whose values are presented in Table 2. Certainly, the measured velocities would be different if a different part was used in the tests. However, the main goal of the present study is to evaluate the variation of the transportation velocity for a single transported item.

As can be seen from the results presented above, the tuning device enables to change considerably the values of the first natural frequency of the conveyor and, as a result, the mean value of the transport velocity, which increases monotonically as the first natural frequency of the conveyor becomes closer to the excitation frequency within the range of positions of the tuning device considered in the experiments.

## 4. Conclusions

The experimental results demonstrate the possibility of obtaining effective transportation by using piezoelectric patches bonded to the leaf springs of vibratory conveyors. Another important conclusion is related to the possibility of designing the vibratory conveyor to enable feeding the piezoelectric actuators directly from the electrical network (thus avoiding the use of driving electronics) and using a simple mechanical tuning device to adjust the transport velocity. Also, the results point toward the potentiality of the piezoelectric driving small or mid-sized industrial vibratory conveyors, which are believed to present a number of advantages as compared to traditional driving systems in terms of energy consumption, vibration transmission, noise emission and maintenance cost. Nonetheless, such aspects need further quantitative evaluation. Finally, it should be pointed-out that although a linear vibratory conveyor was used in the study, piezoelectric driving can be readily applied to bowl feeders as well.

## Figures and Tables

**Table 1.** Characteristics of the components of the prototype.

**Component**	**Material**	**Dimensions (mm)**
Track	Aluminum	Length: 365
		U-shaped cross section: 25.4 × 11.0 × 1.0

Leaf-springs	Aluminum	180.00 × 25.50 × 2.16

Piezoelectric actuators (MIDÉ Quickpack model QP20n)	PZT	50.8 × 25.4 × 0.762

**Table 2.** Values of the first natural frequency of the conveyor and mean transport velocity for different positions of the tuning device.

**Position of the Bolt along the Tuning Device [m]**	**Value of the First Natural Frequency (Hz)**	**Transport Velocity (Mean; Coefficient of Variation) (cm/s; %)**
0.130	53.5	8.24; 1.4
0.125	58.5	7.79; 6.3
0.115	60.2	11.86; 7.6
0.110	62.2	3.80; 1.3
0.095	63.1	2.28; 1.1
0.090	63.3	1.90; 5
0.085	66.6	1.76; 7.9
0.080	73.1	0.94; 7.7
0.075	74.5	0.52; 2.5
0.070	76.8	0.48; 3.3
0.065	77.6	0.45; 6.2
0.060	77.3	0.35; 2.1
